# The role of lacteal integrity and junction transformation in obesity: A promising therapeutic target?

**DOI:** 10.3389/fendo.2022.1007856

**Published:** 2022-11-24

**Authors:** Qingsong Xia, Hui Dong, Yujin Guo, Ke Fang, Meilin Hu, Lijun Xu, Fuer Lu, Jing Gong

**Affiliations:** ^1^ Institute of Integrated Traditional Chinese and Western Medicine, Tongji Hospital, Tongji Medical College, Huazhong University of Science and Technology, Wuhan, Hubei, China; ^2^ Department of Integrated Traditional Chinese and Western Medicine, Tongji Hospital, Tongji Medical College, Huazhong University of Science and Technology, Wuhan, Hubei, China

**Keywords:** lacteal, obesity, button-like junction, zipper-like junction, lipid uptake

## Abstract

Lacteals are the central lymphatic vessels in the villi of the small intestine and perform nutrient absorption, especially dietary lipids, and the transportation of antigen and antigen-presenting cells. Remodeling, proliferation, and cell-cell junctions of lymphatic endothelial cells (LECs) in lacteals are the basis of the maintenance of lacteal integrity and dietary lipid absorption. Normal lipid absorption in the diet depends on sound lacteal development and proliferation, especially integrity maintenance, namely, maintaining the appropriate proportion of button-like and zipper-like junctions. Maintaining the integrity and transforming button-to-zipper junctions in lacteals are strongly connected with obesity, which could be regulated by intestinal flora and molecular signalings, such as vascular endothelial growth factor C-vascular endothelial growth receptor 3 (VEGFC-VEGFR3) signaling, Hippo signaling, Notch signaling, angiopoietin-TIE signaling, VEGF-A/VEGFR2 signaling, and *PROX1*. This manuscript reviews the molecular mechanism of development, integrity maintenance, and junction transformation in lacteal related to obesity.

## Introduction

Obesity is a complex common disease that is associated with genetic background (1), environmental factors (2), and individual behavior (3, 4), challenging global medical and financial burden. The latest data of the NCD Risk Factor Collaboration showed that the worldwide prevalence of obesity has risen from below 1% in 1975 to 6%-8% in 2016. Approximately 2 billion adults were overweight (defined by a body mass index (BMI) of ≥25 kg m^−2^), and 671 million adults were considered to be obese (BMI ≥30 kg m^−2^) (1, 2). The basis of obesity is excessive energy intake over expenditure (5), which is the core of obesity treatment to mediate the balance between energy intake and expenditure (6, 7). The majority of nutrient uptake is absorbed in the small intestine, such as carbohydrates, protein, vitamins, and lipid (8). Of these, the lymphatic vasculature of the intestine plays an essential role in lipid absorption.

The lymphatic vascular system is closely related to immune functions and transporting dietary lipids from the small intestine (9). Lacteals are lymphatic capillaries in the small intestine that absorb dietary lipids and participate in the gut immune response (10). Lacteals have regenerative and proliferative characteristics, distinct from embryonic lymphangiogenesis or quiescent lymphatic vessels in other tissues (11). There are two lacteals in the duodenum and proximal jejunum villi on average, whereas most residual small intestine villi have only one lacteal, and the length of the lacteals shortens gradually with decreased villus length from the proximal duodenum to the distal ileum (11). The lacteal size accounts for 60%-70% of the villus length in different intestinal segments. Currently, anti-obesity drugs have been focused on neuroendocrine mechanisms, including sympathomimetic drugs, 5-HT_2c_ serotonin agonists, opioid receptor antagonist/dopamine, noradrenaline reuptake inhibitor, pancreatic lipase inhibitor, and incretin co-agonists (12), while junction transformation of lacteals in obesity may be a promising target for obesity treatment, appealing to scientists and clinicians (13). Although some recent literatures have summarized the structure, function of lacteal, and molecular mechanism of regulating junctional integrity (14–16), this review covers the development, integrity, and lipid uptake of lacteals, especially exploring the roles of lacteals integrity and junctional transformation focusing on obesity.

## Development and remodeling of lacteals

Prospero homeobox protein 1 (PROX1), lymphatic vessel endothelial hyaluronic acid receptor 1 (LYVE1), C–C motif chemokine 21 (CCL21), neuropilin‐2 (NRP2), and vascular endothelial growth factor receptor 3 (VEGFR3) are the specific markers in lymphatic endothelial cells (LECs) (17). At the embryonic stages of mice, lymphatic vessels start to develop around embryonic day 10 (E10) (18). Prox1-stained cells, marked as a subpopulation of endothelial cells, have been discovered in the early stages of lymphatic vessel development (approximately E9.5-E10.5) (19), and a few lymphatic vessels are detected in intestinal tubes at E13.5-14.5. The formation of the lymphatic branch is activated in the intestine at E16.5, and lymphatic capillaries are formed in intestinal villi around E17.5, accompanied by high expression of VEGFR3 in LECs. In other words, lacteals can be distinguished in the villi at E17.5. There are enough well-integrated LYVE-1^+^ cells in lacteals at E20.5 to absorb lipids from milk after birth, revealing that lacteals in the small intestine mature during the embryonic period (10). Specific deletion of *LYVE-1* in lymphatic vessels of mice induces ablated lacteals and lymph nodes in the small intestine, resulting in dysfunction of lymph drainage, which unveils that the integrity of lacteals plays an essential role in maintaining normal function of lymphatic vessels (20).

Compared with other lymphatic vessels, lacteals sustain remodeling mediated by the vascular endothelial growth factor (VEGF) signaling molecules, which could be produced by small intestinal endothelial cells (11). VEGFs include VEGF-A, VEGF-B, VEGF-C, VEGF-D, VEGF-E, and placental growth factor (PIGF), and there are three receptors, namely, VEGFR-1, VEGFR-2, and VEGFR-3. VEGF-C/VEGFR-3 and VEGF-A/VEGFR-2 regulate lymphatic vessel growth (21, 22). VEGF-C is produced by smooth muscle cells (SMCs) of the intestine and macrophages, playing an essential role in promoting LEC growth, proliferation, and migration *via* the receptor VEGFR-3 (23). In the embryonic stage, the generation of lacteals is dependent on VEGF-C (23), and VEGF-C facilitates lacteal vessels to expand into the intestinal villi to absorb lipids during the postnatal period (10). Furthermore, Notch ligand delta-like protein 4 (DLL4, the downstream mediator of VEGFR3) is the critical signaling molecule of lacteal development (16, 24). A previous study showed that lacteal regeneration relied on the expression of DLL4 (11). In the normal homeostasis of intestinal villi, LEC regeneration is accommodated by VEGF-C signaling and Notch signaling changes.

Immunofluorescence staining showed that a high level of DLL4 was detectable in the tip cells of sprouting lacteals; on the contrary, the lower signal in the stalk cells and residual lacteals (25). The DLL4 expression pattern in lacteals indicates an underlying role of DLL4 in promoting lacteals sprouting. Specifically, deletion of DLL4 in the small intestine of mice led to fewer filopodia [fine cytoplasmic and actin-rich cellular extensions were found in migrating LECs, which indicated ongoing lymphangiogenic response (11)], shorter lacteals, and fewer total LECs than in control mice, which revealed that DLL4 promoted lacteal LEC survival and proliferation (11). Pharmacological blockade of VEGFR3 by a monoclonal antibody against murine VEGFR3 (mF4-31C1) (26) decreased DLL4 protein in lacteals, reduced the number of filopodia, and shortened lacteals in villi of the small intestine. The study also uncovered that VEGFR2 was necessary for filopodia formation and lacteal length to a lesser extent (11). In line with the above research, conditional deletion of VEGFR3 in lacteals reduced the relative lacteal length in the small intestine (24) and the number of *Prox1* ^+^ LECs (27). In mice, the inactivation of VEGFR3 and PI3K subunit p110α induced deficient embryonic mesenteric lymphatic vessels (28). Likewise, deletion of *Pik3r1* (encoding PI3K subunits p85α, p55α, and p50α) induced abnormal lymphatic vessel morphology resulting from defective spouting and maturation of lymphatic vessels (29). *In vitro* study, LECs were isolated from obesity-prone mice and exposed to stearic acid (a long-chain FFA), which decreased VEGFR3 expression, resulting in cell apoptosis and growth inhibition (30). In summary, VEGF-C/D binds to VEGFR2/3 to promote DLL4 expression, and then the DLL4 protein activates the Notch signaling pathway to regulate LEC migration, survival, and sprouting to maintain lacteal regeneration in normal mice.

## Maintenance of lacteal integrity

The connections between LECs have two forms, including zipper-like junctions and button-like junctions. Inconsecutive button-like junctions are distributed among LECs in initial lymphatic vessels, and continuous zipper-like junctions are found in the cell borders of collecting lymphatic vessels downstream (31). Adherens junction proteins (including vascular endothelial cadherin (VE-cadherin), *β*-catenin, p120-catenin) and tight junction proteins (involving occludin, claudin-5, zonula occludens-1, junctional adhesion molecule-A, and endothelial cell-selective adhesion molecule) organize button and zipper junctions (14, 31, 32). VE-cadherin is particularly abundant in button-like junctions (33), which is necessary for the development and maintenance integrity of lymphatic vessels, especially lacteals (31, 34), and deletion of VE-cadherin in mice induced lacteals fragmentation into cysts or highly distended vessels (34). Similarly, tight junction proteins are required to maintain lacteal integrity. For example, apparent lymphatic vessel leakage was observed in *claudin-5* ^+/-^ mice (35). Not only that, junction-related proteins, connexins (a family of transmembrane proteins), seem to be involved in normal lymphatic vessels development and function, especially in lymphatic valves formation, and genetic mutations of connexins induce lymphedema resulting from abnormal lymphatic valve formation (36–38). Nevertheless, other junction-related proteins (e.g., platelet and endothelial cell adhesion molecule 1 (PECAM-1) and LYVE-1) are involved in leukocyte transendothelial migration of immune response, and PECAM-1 is not indispensable for maintenance of junctional integrity (14, 31, 39).

Button-like and zipper-like junctions are vital in maintaining integrity to provide a natural function of lacteals. Both button-like junctions of initial lymphatic vessels and zipper-like junctions of collecting lymphatic vessels are essential for preventing lymph leakage from collecting lymphatic vessels (31, 40). Recent studies unveiled that inconsecutive button-like junctions provided ubiquitous access for interstitial components and immune cells into the lymphatic vessels (15, 31). An investigation revealed that zipper-like junctions converted into button-like junctions in initial lymphatic vessels after birth. Only button-like junctions were observed at ten weeks of age in mice while developing initial lymphatic vessels first had zipper-like junctions in the embryos of mice (41). Several studies uncovered that the underlying mechanism by which zipper-like junctions converted into button-like junctions postnatally might be associated with VE-cadherin regulation by angiopoietin2 (ANG2) and Hippo signaling (40, 42, 43).

Angiopoietin-TIE signaling is essential in regulating lymphangiogenesis and blood vessel formation (44). Angiopoietin 1 (ANG1) and its receptor (TIE2) maintain angiogenesis and remodeling during the embryonic period and promote lymphatic sprouting and growth (45). In comparison, ANG2 is recognized as an antagonist of ANG1/TIE2 signaling, an essential regulator of cell-cell junctions between LECs during lymphatic development, transforming zipper-like junctions into button-like junctions that facilitate this fluid drainage to the lymphatic system in initial lymphatic vessels. ANG2 blockade and deletion reduced zipper-to-button junctional transformation in the initial lymphatic vessels of mice, resulting in peripheral lymphedema, chyle leakage, and defective fluid-draining capacity, which may be related to the inhibition of VE-cadherin phosphorylation at Y685 in the embryonic period, which restrained the transformation into button-like junctions (40).

**Figure 1:**
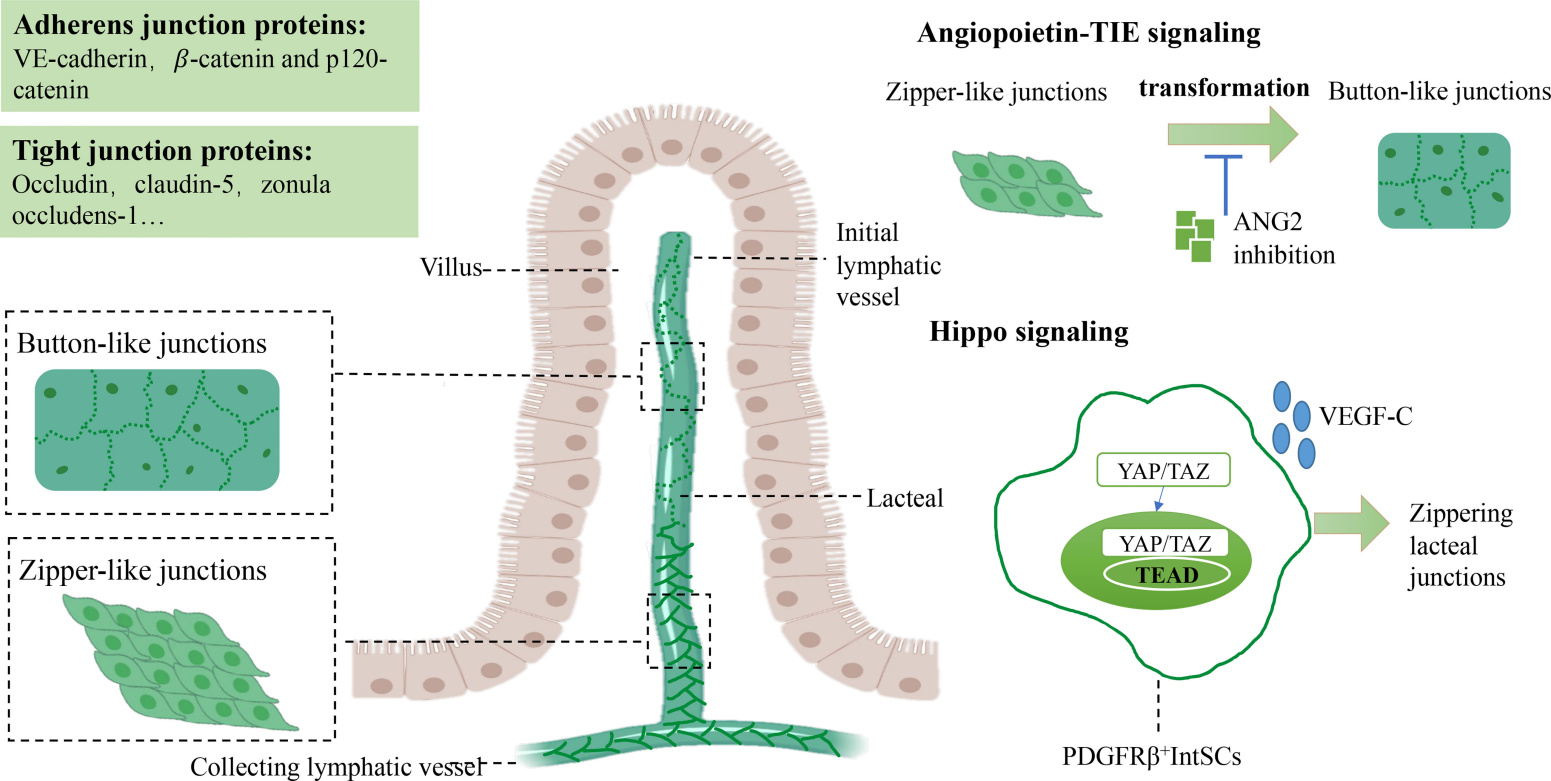
Junctions between LECs and the molecular mechanism of maintaining lacteal integrity. Tight junctions between LECs are composed of button-like junctions and zipper-like junctions in lacteal tissue. Button-like junctions mainly perform lipid absorption functions, whereas zipper-like junctions are found in collecting lymphatic vessels. In angiopoietin-TIE signaling, ANG2 plays a critical role in transforming zipper-like junctions to button-like junctions to maintain normal lipid uptake. The crosstalk between Hippo and VEGF-C/VEGFR3 signaling is essential for mediating the formation of button-like junctions. Elevated zipper-like junctions and increased VEGF-C levels have been observed in YAP/TAZ hyperactivated mice. Vascular endothelial growth factor C, VEGF-C; Vascular endothelial growth factor receptor, VEGFR3; Lymphatic epithelium cell, LEC. TEA domain transcription factors, TEAD.

In addition to Angiopoietin-TIE signaling, Hippo signaling seems to participate in junction transformation. In recent studies, Hippo signaling has been demonstrated to regulate the proliferation and homeostasis of LECs (42, 46, 47). YAP/TAZ is the terminal transcriptional regulator of Hippo signaling, binding TEA domain transcription factors (TEADs) to mediate relative target genes in lymphangiogenesis (42). An exciting study revealed that PDGFRβ+ intestinal stromal cells (IntSCs) maintained lacteal integrity and function by YAP/TAZ-dependent secretion of VEGF-C (43). Hyperactivation of YAP/TAZ decreased the ratio of button-like junctions. In contrast, an enhanced percentage of zipper-like junctions of lacteals resulted in defective lipid absorption, related to activation of YAP/TAZ and subsequent binding to TEADs to upregulate VEGF-C and increase VEGF-C secretion of a subset of fibroblasts after mechanical stimuli (43) (**Figure 1**).

## Lipid uptake in lacteals

The lymphatic system devotes itself to dietary lipid uptake to provide energy to the whole body. Practically, major dietary lipids are transported from the lymphatic system into the blood through chylomicrons in lacteals of the small intestine (13, 48). The proximal duodenum is the uppermost place to absorb dietary lipids hydrolyzed by gastric and pancreatic lipases. Then, the products mix with liver-derived bile acids to comprise free fatty acids (FFAs) monoglyceride micelles (49, 50). Angiopoietin-like 4, a secreted glycoprotein, inhibits lipoprotein lipase and regulates lipid uptake to prevent enterocyte lipid overload in the intestine (51).

### The effect of lacteal contraction on lipid uptake

**Figure 2:**
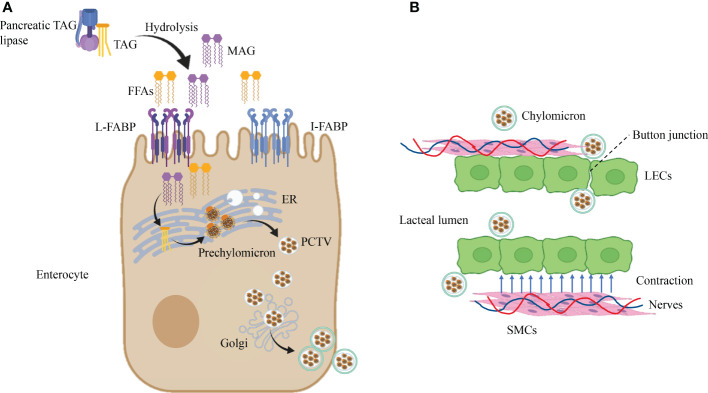
Dietary lipid absorption in lacteals. **(A)** Dietary lipids are incorporated into chylomicrons in enterocytes. Pancreatic TAG lipases convert TAG into FFAs and MAG by the biological process of hydrolysis. Then, the products of hydrolysis are transported in enterocytes after recognition of L-FABP and I-FABP. Subsequently, lipid-synthesizing enzymes turn FFAs and MAG into TAG in the ER, which is incorporated into prechylomicron and embodied in PCTV. Ultimately, after the second incorporation in the Golgi, PCTV is transported near the cell membrane. **(B)** Chylomicron uptake and transportation in lacteal. Chylomicron get access to lacteal lumen through button-like junctions between LECs, and contraction of SMCs close to lacteal provide the power for chylomicron uptake which is controlled by autonomous nervous system. Triacylglycerol, TAG; Free fatty acids, FFAs; FA-binding proteins, FABPs; Sn-2-monoacylglycerol, MAG; Endoplasmic reticulum, ER; Prechylomicron transport vesicle, PCTV.

Dietary lipids are incorporated into chylomicrons in enterocytes. Absorption of FFAs in food requires the assistance of FA-binding proteins (FABPs) to be transported into enterocytes (52). In the small intestine, there are three subtypes of FABP, namely, L-FABP (FABP1), I-FABP (FABP2), and Il-FABP (FABP6), primarily enriched in the proximal intestine (53, 54),which act on lipid transportation and absorption (55, 56). Dietary triacylglycerol (TAG) is hydrolyzed by pancreatic TAG lipase to produce fatty acids (FFAs) and sn-2-monoacylglycerol (MAG), which are absorbed by binding I-FABP and L-FABP in enterocytes. Subsequently, activated by the complex lipid-synthesizing enzymes of the endoplasmic reticulum (ER), FFA and MAG are converted to TAG and are incorporated into prechylomicron in the ER (52, 57). The prechylomicron is embodied in the prechylomicron transport vesicle (PCTV) and transported from the ER membrane to the Golgi. A second transport vesicle carries the prechylomicron to the basolateral membrane, then transported into the lamina propria and finally into the mesenteric lymph by exocytosis (57) (**Figure 2A**). After leaving the basal side of enterocytes by vesicle-mediated transport, mature chylomicrons must pass through the intestinal lamina propria to reach lacteals (58).

At present, button-like junctions of lacteals are recognized as the channel that allows the entry of chylomicron (59, 60). In addition, the transportation of ingoing chylomicron in lacteals needs the assistance of autonomous lacteal contraction. Autonomous lacteal contraction depends on the adjacent smooth muscle cells (SMCs) in the lacteals (61). Autonomous lacteal contraction is accommodated by the autonomic nervous system because these SMCs possess subtypes of cholinergic and adrenergic receptors (62, 63). Acetylcholine significantly increases lacteal contraction, whereas applying atropine and norepinephrine decreases lacteal contraction. Therefore, inhibition of acetylcholine release can lessen lacteal contraction and reduce the assimilation of dietary lipids. Administration of mecamylamine and pentolinium (ganglion-blocking agents) into the mesentery declines lacteal contraction (63). In summary, the coordination of the sympathetic and parasympathetic nervous systems meditates lacteal contraction to regulate the absorption of dietary lipids (**Figure 2B**).

### Regulatory factors of lacteal integrity related to lipid uptake

CD36/FAT: CD36/FAT, expressed in endothelial cells, is the fatty acid transport receptor that recognizes long-chain fatty acids and lipoproteins (64–66). Accumulation of dietary cholesterol in the intestine and reductive dietary cholesterol in lacteals, namely, poor lipid uptake, was observed in *CD36* knockout mice, revealing that CD36 might facilitate cholesterol uptake in the small intestine (67). A recent study showed that *CD36*-specific deletion in LECs of mice that exhibited metabolic switching was associated with insulin resistance and visceral obesity, resulting from disrupted VE-cadherin junctions inducing leaky lacteals. Reduced VEGF-C signaling to VEGFR2/AKT disrupted lacteal integrity and switched metabolic pathways, scilicet inhibition of fatty acid oxidation, and augmented glycolytic rates, which might be the potential molecular mechanism for poor lipid uptake in *CD36* knockout mice (60).

Calcitonin receptor-like receptor (Calcrl)-related signaling: Calcrl, a receptor complex of adrenomedullin (AM), is highly expressed in LECs and is necessary for lacteal integrity and function (68). Dilated lacteals, aberrant chylomicron absorption, and weight loss after a high-fat diet had been found in *Calcrl^-/-^* adult mice, which revealed that maintenance of AM signaling was required for maintaining the integrity and normal function of lipid absorption in lacteals (69). Furthermore, reduced mRNA expression of the relative Notch signaling molecules, namely, Dll4, Hey1, and Notch1, was detected in *Calcrl* knockdown human LECs. Nevertheless, treatment with human AM peptide enhanced the mRNA level of the above molecules, which performed cross-talk between AM signaling and downstream Notch signaling to mediate the integrity and maintain the physiologic function of lacteal cells (68).

Gut microbiota: The gut microbiota is an essential factor that regulates lacteals’ development, maturation, and integrity. Delayed lacteal growth in the postnatal stage, lacteal regression in adulthood, and reduced button-like junctions among LECs had been found in germ-depleted mice. Meanwhile, the depletion of gut microbiota reduced button-like junctions and elevated zipper-like junctions in lacteals, resulting in blocked absorption of dietary fat (27). Thus, balancing the distribution of button-like and zipper-like junctions and maintaining normal lipid uptake in lacteals depends on gut microbiota (70).

**Figure 3:**
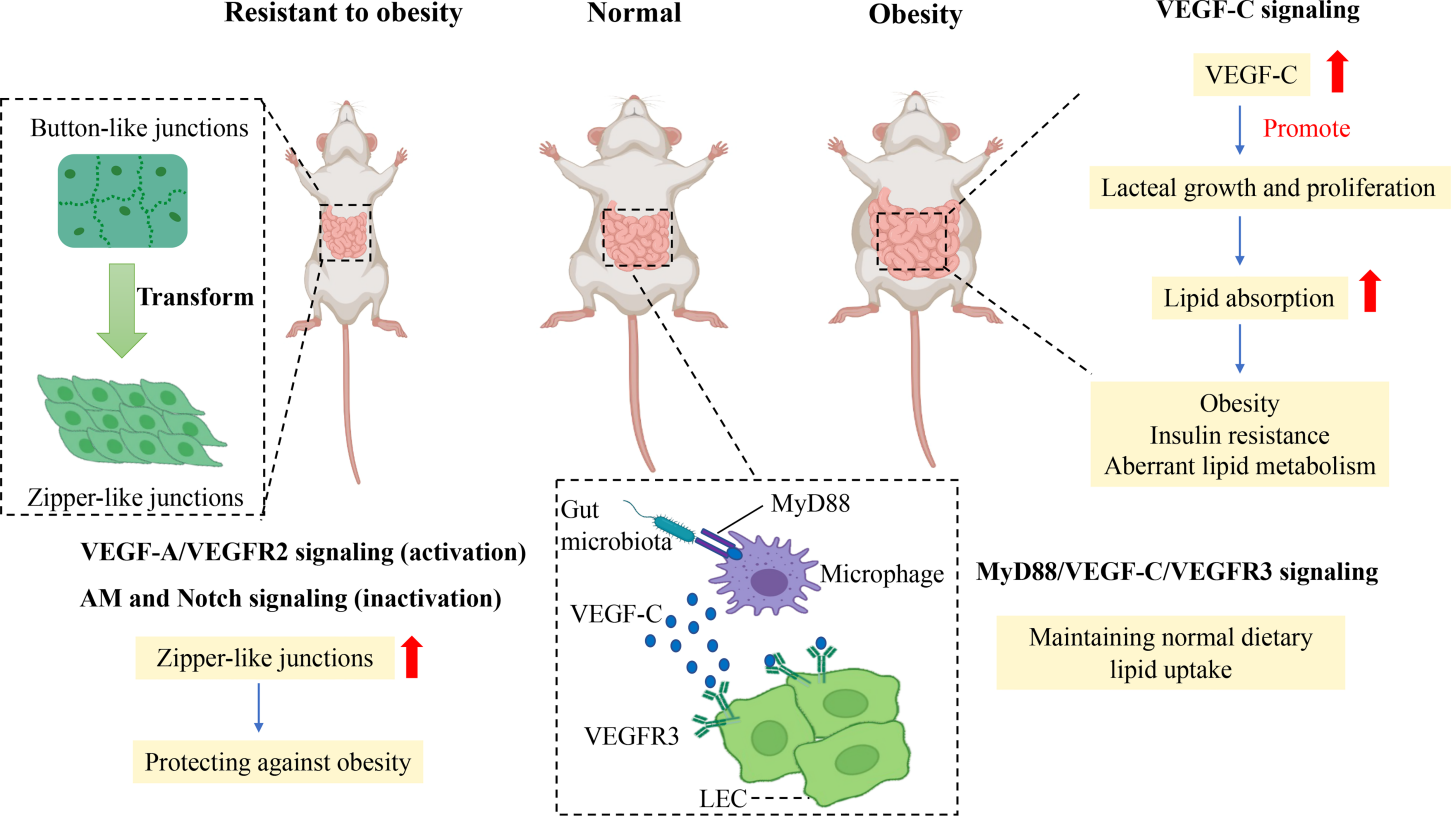
Mechanism of junction transformation and integrity of lacteals related to obesity. Promoting the transformation of button-to-zipper junctions in lacteal LECs is resistant to the obese phenotype of mice, which may be associated with impaired lipid uptake. Activation of VEGF-A/VEGFR2 signaling and inactivation of AM and Notch signaling enhance the proportion of zipper-like junctions of LECs which reduce lipid uptake in the small intestine to prevent obesity. In comparison, the gut microbiota is essential in maintaining normal lipid uptake in lacteals by MyD88/VEGF-C/VEGFR3 signaling. In contrast, the augmented level of VEGF-C promotes lacteal growth and proliferation in obese rodents, which enhances dietary lipid absorption to increase aberrant lipid metabolism, obesity, and insulin resistance. Vascular endothelial growth factor C, VEGF-C; Vascular endothelial growth factor receptor, VEGFR3; Lymphatic epithelium cell, LEC; Myeloid differentiation primary response protein 88, MyD88; Adrenomedullin, AM.

How does gut microbiota mediate lacteal integrity to affect the absorption of dietary fat? VEGF-C protein and the number of macrophages were reduced in the jejunum and ileum of germ-depleted mice, indicating that macrophages were indispensable for lacteal integrity. Likewise, decreased VEGFR3 expression and *Prox1* ^+^ LECs numbers were visible in lacteals of germ-free (GF) mice (27). Furthermore, depletion of CX3CR1^+^ villus macrophages in CX3CR1-DTR mice significantly diminished button-like junctions and the length of lacteals, VEGF-C mRNA level, VEGFR3 expression, and the number of *Prox1* ^+^ LECs, which indicated that villus macrophages were the critical factor for the maintenance of lacteal integrity (27). Myeloid differentiation primary response protein 88 (MyD88) is an essential signal transduction molecule for macrophages to recognize gut microbiota and their product (71). Conditional deletion of MyD88 in villus macrophages reduced the mRNA level of VEGF-C, the proportion of button junctions of lacteals, the number of *Prox1* ^+^ LECs, and VEGFR3 expression in the small intestine (27). Therefore, the intestinal microbiota mediates lacteal integrity and lipid uptake by MyD88/VEGF-C/VEGFR3 signaling (**Figure 3**). Generally, lipid uptake relies on lacteal integrity, which is associated with CD36/FAT, Calcrl-related signaling, and gut microbiota.

Besides, recent studies have found that VEGF-C overexpression is detrimental to metabolism in adulthood, although VEGF-C is essential for developing LECs. A study showed reduced lipid absorption and resistance to HFD-induced obesity in adult mice if adult mice were chronically deleted the *Vegfc* gene using the Cre-Lox system (24). Likewise, a study showed that K14–VEGFR-3–Ig(sR3) mice that expressed soluble VEGFR-3–Ig in the skin to scavenge VEGF-C were protected against HFD-induced insulin resistance and hepatic lipid accumulation, which was related to an increased M2/M1 macrophage ratio (72). In contrast, significantly elevated VEGF-C mRNA in subcutaneous adipose tissue of HFD-induced obese mice was observed in a recent study (72), and increased serum VEGF-C was related to insulin resistance and aberrant lipid metabolism in individuals with obesity in a cross study (73). In addition, VEGF-C overexpression in transgenic mice increased weight gain and subcutaneous adipose tissue accumulation and showed metabolic deterioration, such as insulin resistance and increased ectopic lipid accumulation (74). Thus, overexpression of VEGF-C can reduce insulin sensitivity and accelerate obesity-induced insulin resistance and aberrant lipid metabolism. In other words, controlling VEGF-C expression may be a potential target to treat obesity and insulin resistance (**Figure 3**).

## The effect of junction transformation and lacteal integrity on obesity

### The transformation of button-like junction to zipper-like junction may be a novel target to mitigate obesity

Button-like junctions provide convenient access to permit chylomicron to pass in lacteals when the body assimilates lipids from food. Increasing zipper-like junctions in lacteals seems to be an ideal approach to alleviate HFD-induced obesity. A recent study showed that vascular endothelial growth factor receptor 1 (FLT1) and neuropilin1 (NRP1) deletion increased zipper-like junctions and impeded dietary lipid uptake, resulting in resistance to HFD-induced obesity. The molecular mechanism was that the absence of NRP1 and FLT1 receptors bumped up VEGF-A bioactivity and signaling through VEGFR2, accelerating lacteal junction zippering and defective chylomicron uptake. High VEGF-A signaling transformed button-like junctions into zipper-like junctions, making lacteal junctions more compact concomitantly with reduced lipid absorption to prevent mice from HFD-induced obesity (59).

In accordance with the above study, activation of VEGF-A/VEGFR2 signaling promotes the transformation of button-to-zipper junctions to reduce lipid uptake, which is resistant to obesity (59, 60, 67). In contrast, VEGF-C, released by macrophages under the stimulation of gut microbiota and its products, activates VEGFR3 to increase button-like junctions to promote lipid uptake in lacteal tissue (27). Besides VEGFR signaling, Notch signaling also is attributed to mediating junction transformation in obesity.

DLL4 is essential in maintaining LEC growth and regeneration and regulating lipid uptake. Specially knocking out *DLL4* at the gene level induced continuous VE-cadherin junctions in LECs and significantly impaired lipid uptake and transportation in lacteal tissue (11). The inactivation of DLL4 reduced lipid uptake resulting from enhancing zipper-like junctions. Furthermore, the crosstalk between Notch signaling and calcrl-AM signaling regulates lipid uptake and junctions of LECs in lacteal. Calcrl, an upstream regulator of DLL4, is responsible for maintaining normal LEC junctions to conduct lipid absorption. No DLL4 expression, more continuous VE-cadherin junctions, and impaired lipid uptake of lacteals were discovered in *Calcrl-*null mice compared with those in control mice (68). Therefore, the inactivation of AM signaling and Notch signaling enhances the proportion of zipper-like junctions of lacteals to protect against obesity.

The transformation between button-like junctions and zipper-like junctions controls lipid uptake in lacteals, which relies on regulating VEGF-A/VEGFR2 signaling, MyD88/VEGF-C/VEGFR3 signaling, Notch signaling, and AM signaling. In general, lipid uptake depends on the transformation between the button-like and zipper-like junction, which may be a potential treatment strategy for obesity (**Figure 3**).

### The impact of lacteal integrity on obesity

Lacteal integrity is intimately involved in button-like and zipper-like junctions to maintain normal lipid uptake. VEGFR2/AKT inactivation by deletion of *CD36* disrupts lacteal integrity and induces lymph leakage, resulting in visceral adiposity (60). High VEGF-A signaling by deletion of *NRP1* and *FLT1* increases zipper-like junctions to make lacteals tighter, lipid uptake lower, which protects against obesity (59). Low AM and Notch signaling are a characteristic of defective chylomicron uptake and weight loss resulting from the damaged integrity of lacteals (68). In addition, villus macrophages recognize gut microbiota and their products depending on MyD88/VEGF-C/VEGFR3 signaling to regulate button-like junctions among LECs, maintain lacteal integrity and affect dietary lipid absorption (27).

*Prox1* is an essential gene that regulates the development of LECs (19). Similarly, the *Prox1* gene is linked with lacteal integrity resulting in obesity. *Prox1* ^+/-^ transgenic mice with impaired lymphatic function showed an obese phenotype that accumulates subcutaneous and visceral adipose tissue, resulting from chyle leakage (the lipid-rich fluid transported by lacteals) from defective lymphatic vessels (75, 76). The obese phenotype of *Prox1* ^+/-^ heterozygous mice, namely, aberrant hepatic lipid accumulation and lipid accumulation in the small intestinal wall, is attributed to defective lacteal integrity and leakage of chyle during lymph transport. Interestingly, the *Prox1* gene seems to be associated with human obesity and type 2 diabetes mellitus (T2DM) in clinical studies. A European cross-sectional study showed that single nucleotide polymorphisms (SNPs) in *Prox1* were involved in genetic susceptibility to T2DM (77). In another genome-wide association study, body mass index and waist circumference were strongly correlated with SNPs in the *Prox1* gene from 1049 Mongolian individuals, revealing that the 1q32 and 10q11.22 loci near the *Prox1* gene were new candidate loci for obesity (78). However, the relevance of the *Prox1* SNPs to lacteal integrity in humans is still unclear.

## Conclusion

Lacteal pathophysiology, including development, lacteal integrity, cell-cell junctions, and the underlying molecular mechanisms of junction transformation and integrity of lacteals related to obesity, were summarized here. The small intestine has a powerful function in lipid absorption and immunosurveillance of gut microbiota, mainly depending on lacteals (15). During the development of lacteal tissue, VEGF-C/D-VEGFR2/3 signaling and Notch signaling play an essential role in lacteal spouting and regeneration. Maintaining lacteal integrity depends on ANG2-TIE signaling and Hippo signaling. Hyperactivation of YAP reduced button-like junctions and impeded the transport of chylomicron into lacteals, resulting in impaired lipid absorption (43). Notably, an obese phenotype was observed in *Prox1* ^+/-^ heterozygous mice. Interestingly, the SNPs of *Prox1* showed a solid relation to obesity and T2DM in a human study.

Lipid uptake is closely related to the cell-cell junctions of LECs, and VEGF-A/VEGFR2 signaling, AM signaling, and VEGF-C signaling regulate the formation of junctions in lacteals to absorb lipids. Activation of VEGF-A/VEGFR2 signaling and inactivation of AM-Notch signaling can enhance the transformation of button-to-zipper junctions, inducing obesity resistance. In contrast, VEGF-C overexpression increases body weight and accelerates aberrant lipid metabolism, resulting in obesity and insulin resistance. Currently, anti-obesity medications confront various challenges, such as heterogeneity of patient cohorts and safety, and the novel drug candidates on basis of several promising therapeutic targets (leptin, incretin, ghrelin, mitochondrial uncoupler, and growth differentiation factor 15) have been advanced to human trials (12). Regulating the transformation of button-to-zipper junctions in lacteal tissue may be a potential strategy to treat obesity. Nevertheless, it is still unknown whether the above signaling pathways can regulate junctions and lipid uptake of lacteals in humans to treat obesity.

In the clinic, lacteal dysfunction results in defective nutrient absorption, such as protein-loss enteropathy-induced systemic lymphopenia, hypoalbuminemia, and hypogammaglobulinemia, because of serum protein loss in the gastrointestinal tract (79). In familiar clinical inflammatory bowel diseases, Crohn’s disease is thought to be related to intestinal lymphangiectasia with increased intestinal lymphatic capillary permeability during inflammation (80). In colorectal cancer (CRC) patients without distant metastases, the status and number of lymph nodes are associated with disease outcome and patient survival (81). Studies have shown that VEGF-C overexpression multiplied tumor lymphangiogenesis and lymph node metastasis in orthotopic models of CRC, which could be blocked by VEGFR3-blocking antibodies (82, 83).

VEGF-C signaling, the transformation of zipper-to-button junctions, and *Prox1* have been confirmed to be related to obesity. Enhancing the transformation of button-to-zipper junctions and attenuating VEGF-C levels have been demonstrated to resist obesity in mouse models. The small intestine is a large endocrine organ that synthesizes and secretes hormones, such as peptide YY, glucagon-like peptide 1 (GLP-1), cholecystokinin, somatostatin, gastric inhibitory peptide, serotonin, and ghrelin, which function to regulate metabolism, namely, insulin secretion, nutrient absorption and food intake (84, 85). Gut hormones have formed the basis of existing clinical treatments for T2DM and obesity (85). Is dietary fat absorption in lacteals associated with intestinal endocrine hormones to regulate obesity and T2DM? If we determine this, promising methods might be found to prevent and treat obesity and T2DM.

## Author contributions

QX and YG, writing - original draft, writing – review, and editing. MH and KF, visualization. HD and LX, resources and investigation. JG and FL, supervision and conceptualization. All authors contributed to the article and approved the submitted version.

## Funding

This study was supported by the National Natural Science Foundation of China, NO. 81904158, NO.82004200, NO. 81904010, and the National Key R&D Program of China, NO. 2018YFC1704200.

## Acknowledgments

Most icons in the figures are supported by Biorender, and the article has got access to the publication and licensing rights of Biorender.

## Conflict of interest

The authors declare that the research was conducted in the absence of any commercial or financial relationships that could be construed as a potential conflict of interest.

## Publisher’s note

All claims expressed in this article are solely those of the authors and do not necessarily represent those of their affiliated organizations, or those of the publisher, the editors and the reviewers. Any product that may be evaluated in this article, or claim that may be made by its manufacturer, is not guaranteed or endorsed by the publisher.
